# Integration of basement membrane-related genes in a risk signature for prognosis in clear cell renal cell carcinoma

**DOI:** 10.1038/s41598-024-54073-1

**Published:** 2024-02-16

**Authors:** Bowen Xia, Jingwei Wang, Dongxu Zhang, Xiaopeng Hu

**Affiliations:** 1 Department of Urology, Beijing Chao-Yang Hospital, Capital Medical University, No. 8 Worker’s Stadium, Chaoyang District, Beijing, 100020 China; 2 Institute of Urology, Capital Medical University, Beijing, China; 3 Department of Occupational Medicine and Toxicology, Clinical Center for Interstitial Lung Diseases, Beijing Institute of Respiratory Medicine, Beijing Chaoyang Hospital, Capital Medical University, Beijing, 100020 China

**Keywords:** Renal clear cell carcinoma, Basement membrane-related genes, Basement membrane, Gene signature, Prognosis, Cancer screening, Biomarkers, Risk factors, Tumour biomarkers

## Abstract

Clear cell renal cell carcinoma (ccRCC) is characterized by high heterogeneity and recurrence rates, posing significant challenges for stratification and treatment. Basement membrane-related genes (BMGs) play a crucial role in tumor initiation and progression. Clinical and transcriptomic data of ccRCC patients were extracted from TCGA and GEO databases. We employed univariate regression and LASSO-Cox stepwise regression analysis to construct a BMscore model based on BMGs expression level. A nomogram combining clinical features and BMscore was constructed to predict individual survival probabilities. Further enrichment analysis and immune-related analysis were conducted to explore the enriched pathways and immune features associated with BMGs. High-risk individuals predicted by BMscore exhibited poorer overall survival, which was consistent with the validation dataset. BMscore was identified as an independent risk factor for ccRCC. Functional analysis revealed that BMGs were related to cell–matrix and tumor-associated signaling pathways. Immune profiling suggests that BMGs play a key role in immune interactions and the tumor microenvironment. BMGs serve as a novel prognostic predictor for ccRCC and play a role in the immune microenvironment and treatment response. Targeting the BM may represent an alternative therapeutic approach for ccRCC.

## Introduction

Renal cancer is among the most commonly diagnosed types of tumors in the urological system. According to the statistical data from 2020, there would be an estimated 430,000 new cases and 180,000 new deaths worldwide^1^. Renal cancer exhibits a diverse range of histological subtypes, with clear cell renal cell carcinoma (ccRCC) being the most prevalent subtype, accounting for approximately 70% of malignant kidney tumors^2^. ccRCC displays highly complex biological behavior, characterized by high recurrence, metastasis rates, and mortality, significantly impacting patient prognosis^2–4^. With the expanding and deepening understanding of tumor molecular mechanisms, researchers have identified numerous molecular biomarkers that can be used for the diagnosis and prognostic assessment of ccRCC^5^. Biomarkers with high accuracy, specificity, and sensitivity can provide significant assistance in the diagnosis, treatment, and prognostic evaluation of ccRCC patients.

The basement membrane (BM) is a dense layer of extracellular matrix in animals, composed of various components, primarily consisting of collagen-IV and laminin^6^. BM acts with a mechanical stress resistance barrier, determines tissue shape, and creates a diffusion barrier^7^. The BM separates epithelial, endothelial, neural, and adipose cells from underlying tissues, playing a crucial role in tissue architecture, cell signaling, and barrier function integrity^8^. The diversity and fundamental functions of over 200 BM-related genes (BMGs) underscore their significance as the foundation for human diseases^9^. For instance, BM proteins serve as targets for autoantibodies in immune-related disorders^10^. Additionally, defects in BM protein expression and turnover contribute to pathogenic factors in cancer, diabetes, and fibrosis^11,12^. Invasion of the BM is a crucial process in tumor invasion and metastasis. Based on the relationship between renal tumor cells and the BM, renal cancer can be classified into pre-invasive lesions and invasive lesions, typically associated with distinct prognostic outcomes^13^.

In carcinoma, tumor cells invade the BM, lymph nodes, and blood vessels, leading to metastasis^14^. The integrity of BM function and BMGs have been identified as crucial prognostic indicators in lung cancer, liver cancer, breast cancer, and bladder cancer^15–18^. Matrix metalloproteinases (MMPs), which can degrade the main components of BM^19^, have been reported to have a significant correlation with tumor staging in renal cancer, particularly an increased expression of MMP9^20^. Furthermore, other proteases capable of disrupting BM integrity have been found to be significantly upregulated in ccRCC, suggesting the silencing of BMGs as a potential therapeutic target for ccRCC^21^. Unfortunately, there is limited research integrating BMGs for comprehensive analysis and defining their clinical value in the prognosis of ccRCC patients. Accurate BM-related features would be highly valuable for predicting the prognosis of ccRCC and improving treatment outcomes.

In this study, we investigated the association among BMGs and ccRCC prognosis, immune microenvironment, and treatment based on high-throughput expression profiling data related to ccRCC. Firstly, we performed subtyping analysis of ccRCC tumor samples based on the expression levels of BMGs. Subsequently, we further identified prognostic-related BMGs and constructed a prognostic risk prediction model using these genes. The model was validated internally and externally. Finally, we analyzed the immune cell characteristics and functional enrichment in different risk groups.

## Results

### Analysis of ccRCC subtypes based on BMGs

**Figure 1:**
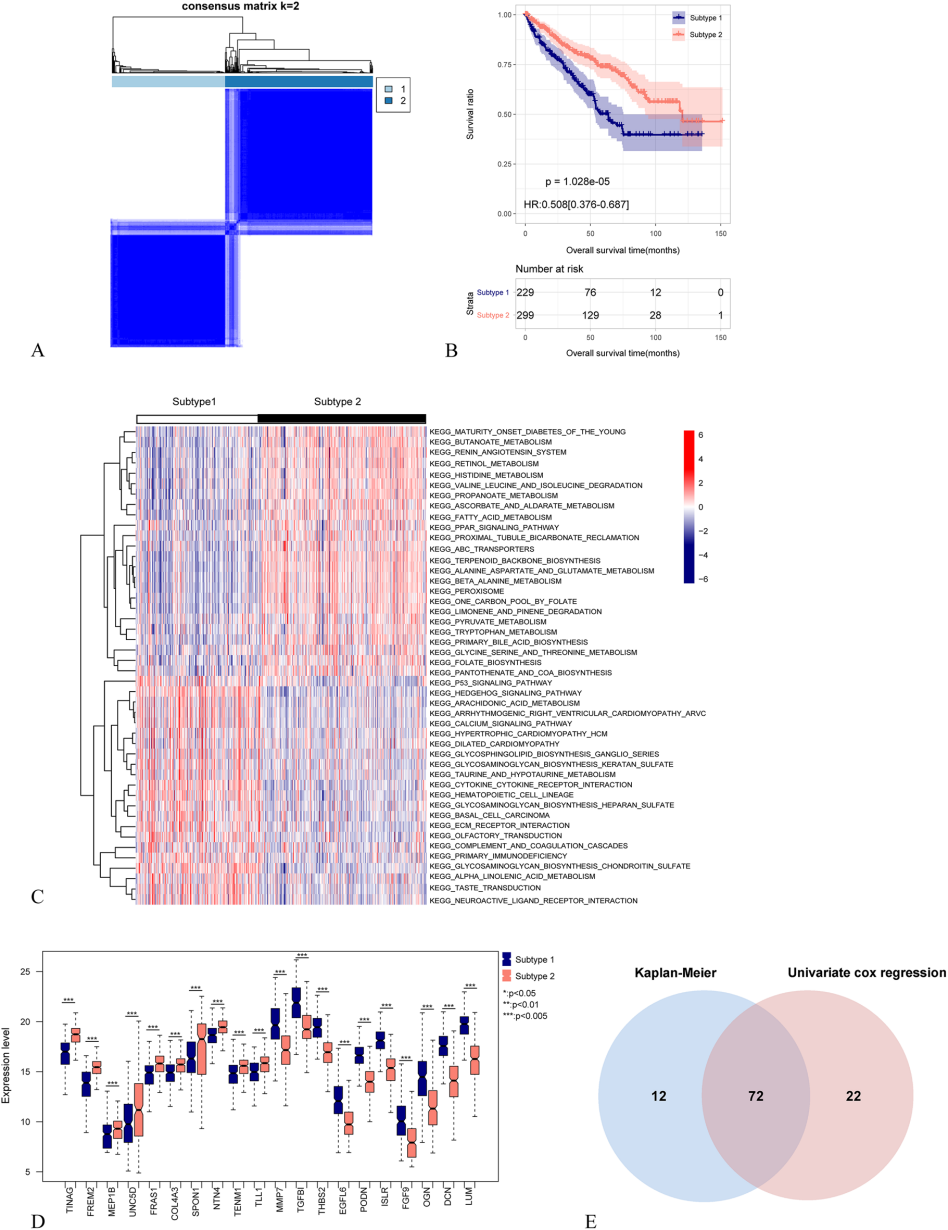
Based on the analysis of BMGs in ccRCC and the identification of prognostic-related BMGs. (**A**) Hierarchical clustering analysis of ccRCC samples based on BMGs. (**B**) Kaplan–Meier survival curves for different subtypes. (**C**) Display of KEGG signaling pathways with significant distribution differences among different subtypes. (**D**) Top ten significantly differentially expressed BMGs among different subtypes. (**E**) Comparative Venn diagram of prognostic-related BMGs identified through KM and univariate Cox regression analysis, resulting in the identification of 72 prognostic-related BMGs.

The workflow of the analysis process was shown in Fig. S1. Based on the expression levels of 222 BMGs in KIRC samples, a disease subtyping analysis was performed. As shown in Fig. 1A, two distinct and evident subtypes, Subtype 1 and Subtype 2, were identified, consisting of 229 and 299 ccRCC samples, respectively. Kaplan–Meier method was used to assess survival prognostic differences between different disease subtypes. The results demonstrated significantly different survival prognostic profiles between the subtypes, with the subtype 1 group exhibiting worse clinical outcomes (Fig. 1B).

To compare the differences in KEGG signaling pathways and the expression levels of BMGs among different subtypes, we identified a total of 46 significantly differentially distributed KEGG pathways (Fig. 1C) and 178 significantly differentially distributed BMGs (Fig. 1D, with the top 10 upregulated and top 10 downregulated genes selected based on fold change differences).

### Construction of BMGs-related prognostic risk prediction model and efficacy assessment

Based on the identified 178 BMGs with significant expression differences in ccRCC subtypes, 84 and 94 prognosis-related genes were screened using the KM method and univariate cox regression, respectively. Among these, 72 genes were found to be overlapping in both gene sets (Fig. 1E). PPI network profiling of the 72 BM genes revealed 14 significantly correlated KEGG signaling pathways (Fig. S2A,B). Further regression analysis using the LASSO algorithm was performed on the 72 prognostic-related genes set (Fig. S3) to identify the optimal combination of relevant genes. Consequently, a total of 12 gene combinations (ACHE, ADAMTS14, COL4A4, COL9A3, FREM2, GPC4, ITGA6, ITGA9, MATN4, MUSK, P3H1, TIMP3) were obtained. Furthermore, the risk-associated BM score was calculated for each patient in training cohort.

**Figure 2:**
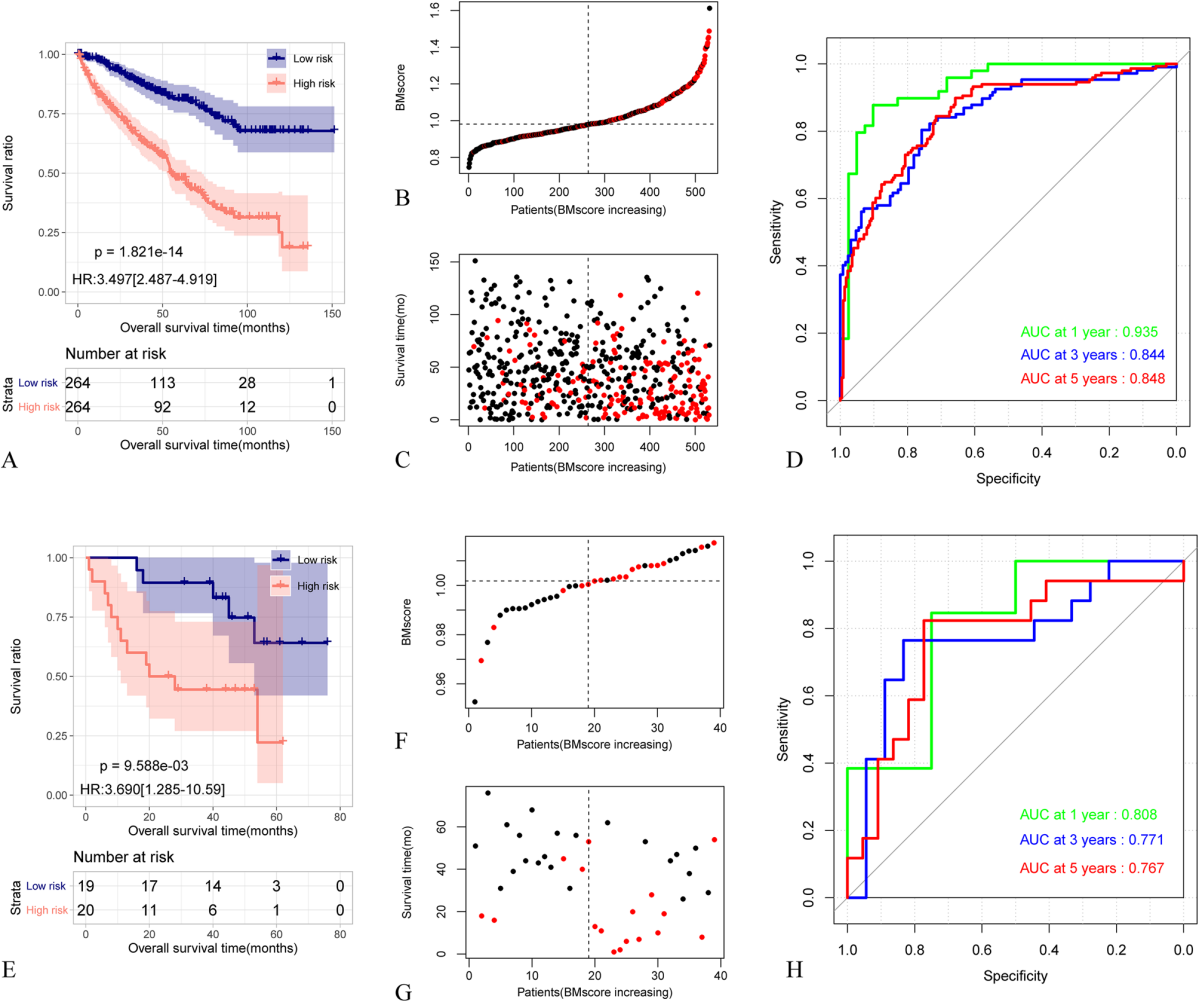
Construction of a prognostic risk prediction model based on BMGs and its performance evaluation. (**A**) Kaplan–Meier curves related to the prognostic model based on 12 optimal BMGs in the training set. The blue and red curves represent the low-risk and high-risk sample groups, respectively. (**B**,**C**) Distribution of BMscores and survival status in the training set, black dots indicate a state of Live, and red indicates Death. (**D**) ROC curves at 1, 3, and 5 years based on the prognostic model of 12 optimal BMGs in the training set. (**E**) Kaplan–Meier curves related to the prognostic model based on 12 optimal BMGs in the validation set. The blue and red curves represent the low-risk and high-risk sample groups, respectively. (**F**,**G**) Distribution of BMscores and survival status in the validation set, black dots indicate a state of Live, and red indicates Death. (**H**) ROC curves at 1, 3, and 5 years based on the prognostic model of 12 optimal BMGs in the validation set.

The BMscore was calculated for individual samples in the training and validation sets. According to the median BM scores, the samples in the training set and the GSE29609 validation dataset are subdivided into 2 groups with high and low BMscores, respectively. Correlations between BM score subgroups and clinicopathologic variables were summarized in Table 1. In Fig. 2B,C and F,G, the BMscore distributions and survival status of the training and validation cohorts are shown, a progressive increase in mortality was observed in both cohorts with higher BMscore levels. The effectiveness of BM score grouping in predicting ccRCC prognosis was evaluated using Kaplan–Meier analysis. The outcomes demonstrated that samples in the high BMscore group had significantly worse overall survival (OS) versus those in the low BMsocre group in both the training and the validation cohorts (Fig. 2A,E). The area under the curve (AUC) for the OS curves of BM score at 1-year, 3-year, and 5-year intervals were 0.935, 0.844, and 0.848, respectively, in the training set. In the validation cohort, the AUC values were 0.808, 0.771 and 0.767 (Fig. 2D,H). These results indicate that BM score can effectively predict the prognosis of ccRCC. The results of immunohistochemical analysis indicate that the expression levels of COL9A3, GPC4, and ITGA6 in renal cancer tissue are higher than those in normal tissue, whereas the expression levels of FREM2, ITGA9, and P3HI are lower than in normal tissue (Fig. S4). The immunohistochemical results for the remaining six proteins are not available in The Human Protein Atlas.

**Table 1:** BMGs-related BM score KIRC clinicopathological factors.

Characteristics	BM score		P-value
	Low (n = 264)	High (n = 264)	
Age			0.931
≤ 60	131	133	
> 60	133	131	
Gender			**0.003**
Male	155	189	
Female	109	75	
T_stage			**< 0.001**
T1	169	100	
T2	31	38	
T3	63	116	
T4	1	10	
N_stage			**0.015**
N0	115	124	
N1	3	13	
Nx	146	127	
M_stage			**< 0.001**
M0	233	187	
M1	17	61	
Mx	14	16	
Pathologic_stage			**< 0.001**
Stage I	167	96	
Stage II	28	29	
Stage III	50	73	
Stage IV	19	66	
Neoplasm_histologic_grade			**< 0.001**
G1	12	1	
G2	147	80	
G3	84	121	
G4	14	61	
Gx	7	1	

Bold indicates statistically significant values.

### Identified independent survival prognosis factors and establishment of clinicopathologic nomogram

**Figure 3:**
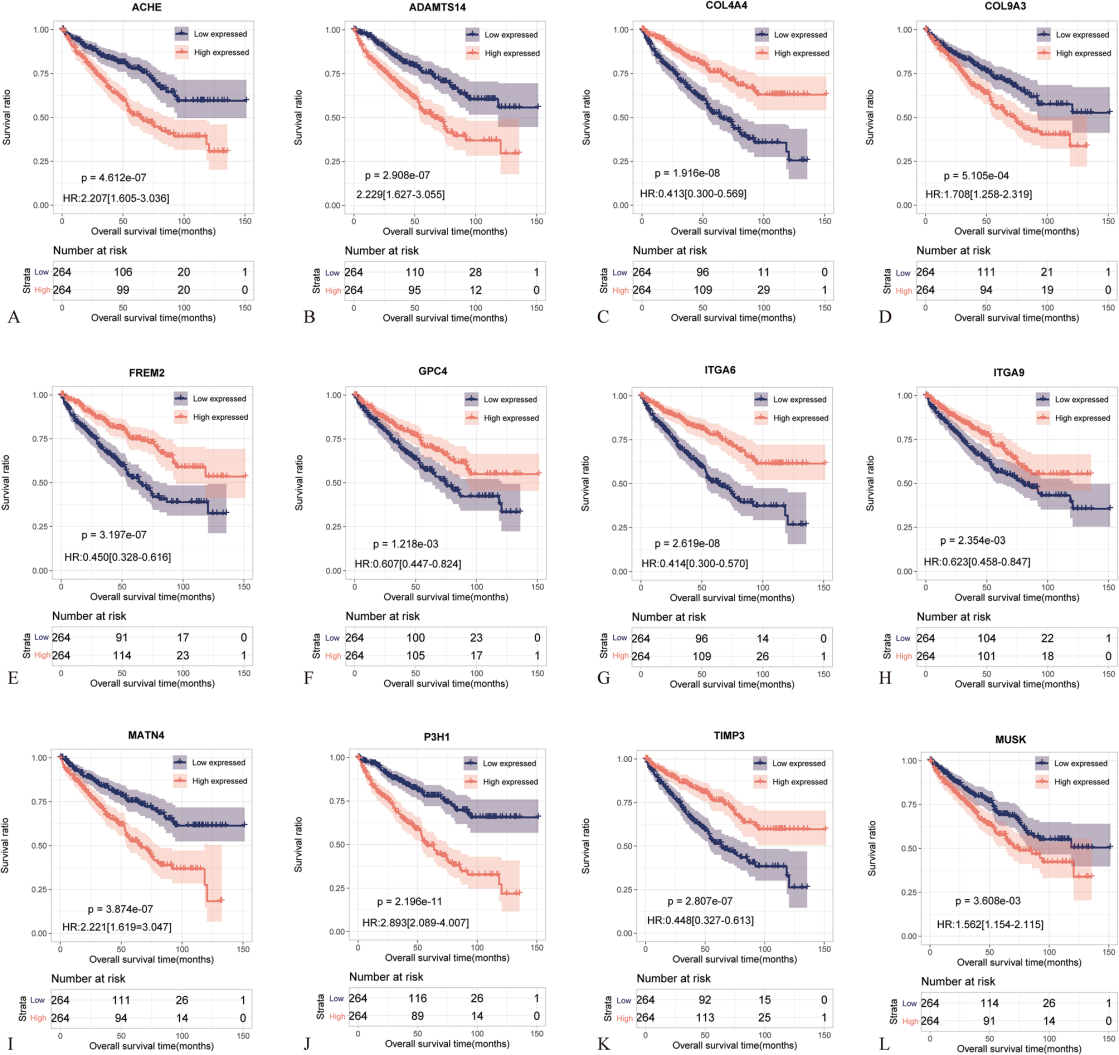
Kaplan–Meier curves related to the prognostic significance of 12 optimized BMGs. The blue and red curves represent the low-expression and high-expression groups. (**A**) ACHE, (**B**) ADAMTS14, (**C**) COL4A4, (**D**) COL9A3, (**E**) FREM2, (**F**) GPC4, (**G**) ITGA6, (**H**) ITGA9, (**I**) MATN4, (**J**) P3H1, (**K**) TIMP3, (**L**) MUSK.

As shown in Fig. 3A–L, all genes comprising the BM score were significantly associated with survival prognosis. Differences in expression levels of BM score-associated genes among BM score groups are illustrated in Fig. S5. Through exploration discovery using the public database GEPIA, compared with normal kidney tissues, COL4A4, COL9A3, FREM2, and P3H1 exhibited significantly different expression levels in ccRCC patients (Fig. S6). As for the survival related data, all BM score related BMGs correlated significantly with patient DFS except COL9A3, GPC4, and ITGA9 (Fig. S7). The expression profile of the BMGs signature in normal tissues and other tumor tissues is depicted in Fig. S8.

**Figure 4:**
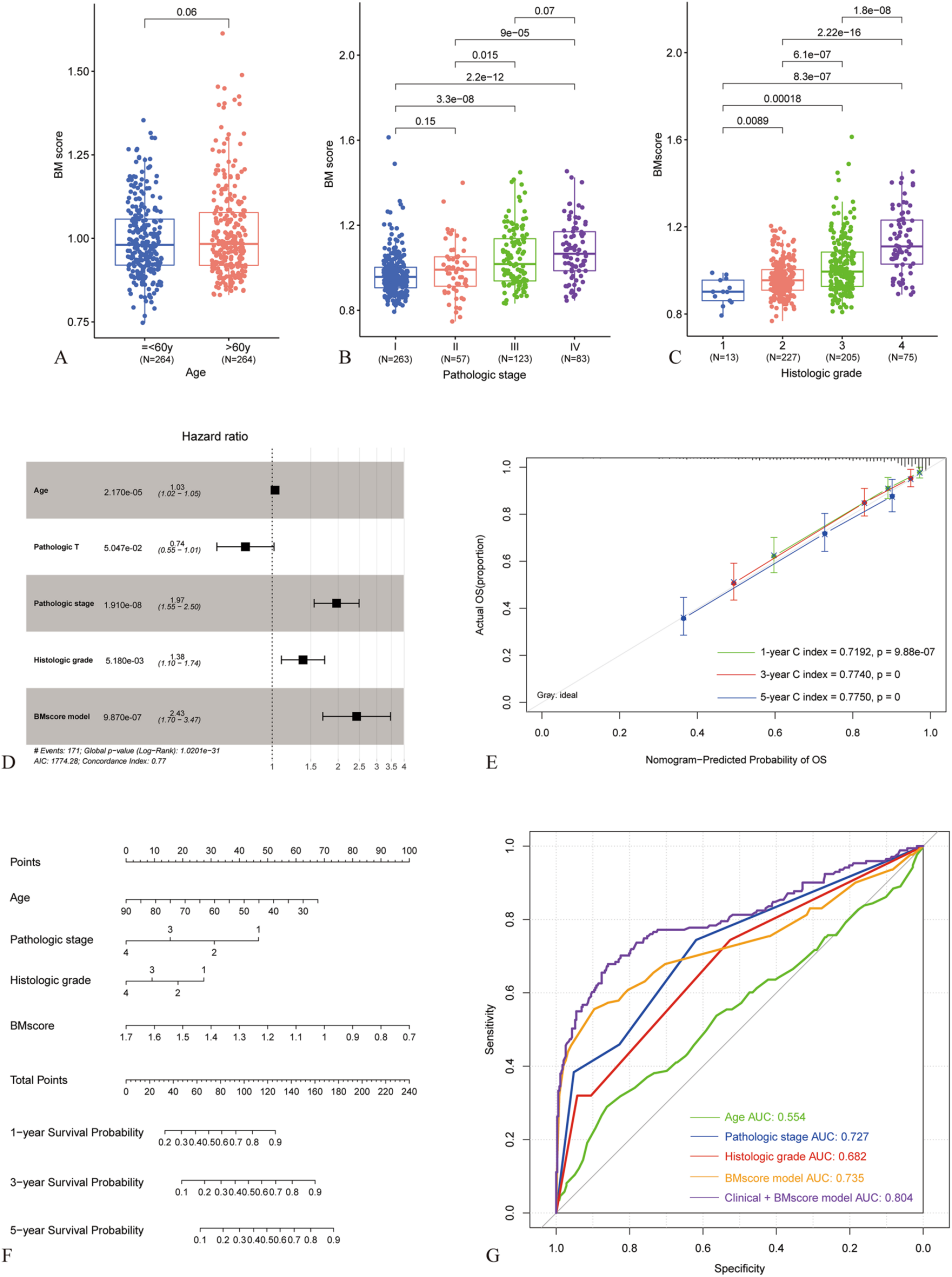
Identification of independent prognostic factors and construction of a nomogram survival rate model. (**A**–**C**) Selection of independent prognostic clinical factors: BMscores levels in different groups based on Age, Pathologic stage, and Neoplasm histologic grade. (**D**) Forest plot displaying independent prognostic factors. (**E**) Concordance plot showing the consistency between predicted and actual survival rates at 1, 3, and 5 years. The x-axis represents the predicted survival rates, and the y-axis represents the actual survival rates. (**F**) Nomogram plot of the prognostic model incorporating BMscore and clinical factors as independent prognostic factors. (**G**) ROC curves for the four independent prognostic factors.

In the clinical information of the samples, the distribution of BM score in different groups of age, pathologic stage, and neoplasm histologic grade is shown in Fig. 4A–C. There were statistically significant differences in BM score between different groups, with an increase in BM score observed with increasing age, pathologic stage, and neoplasm histologic grade. To construct a predictive model for ccRCC survival rates, univariate and multivariate Cox regression analyses were performed on the clinical information of the training set samples. The outcomes were shown in Table 2 and Fig. 4D, revealing four independent prognostic factors: age, pathologic stage, neoplasm histologic grade, and BM score. Based on the above analysis results, we developed accurate 1-year, 3-year, and 5-year OS nomogram based on BMG expression to evaluate ccRCC prognosis (Fig. 4E,F). The AUC value of this model was 0.804, while the AUC value of the model using BM score alone was 0.735 (Fig. 4G).

**Table 2:** Clinical prognostic factors prognostic correlation.

Clinical characteristics	Uni-variable cox		Multi-variable cox	
	HR [95% CI]	P value	HR [95% CI]	P value
Age	1.029 [1.016–1.042]	**< 0.001**	1.030 [1.016–1.045]	**< 0.001**
Gender	0.950 [0.697–1.295]	0.745	–	–
Pathologic M	4.320 [0.961–5.904]	0.081	–	–
Pathologic N	3.414 [0.812–6.434]	0.053	–	–
Pathologic T	1.918 [1.628–2.260]	**< 0.001**	0.741 [0.549–1.001]	0.054
Pathologic stage	1.884 [1.652–2.150]	**< 0.001**	1.967 [1.554–2.490]	**< 0.001**
Neoplasm histologic grade	2.304 [1.880–2.824]	**< 0.001**	1.383 [1.102–1.736]	**0.005**
BMscore model	3.497 [2.487–4.919]	**< 0.001**	2.430 [1.703–3.468]	**< 0.001**

Bold indicates statistically significant values.

### Identified and functionally enriched analysis of DEGs based on BM score grouping

**Figure 5:**
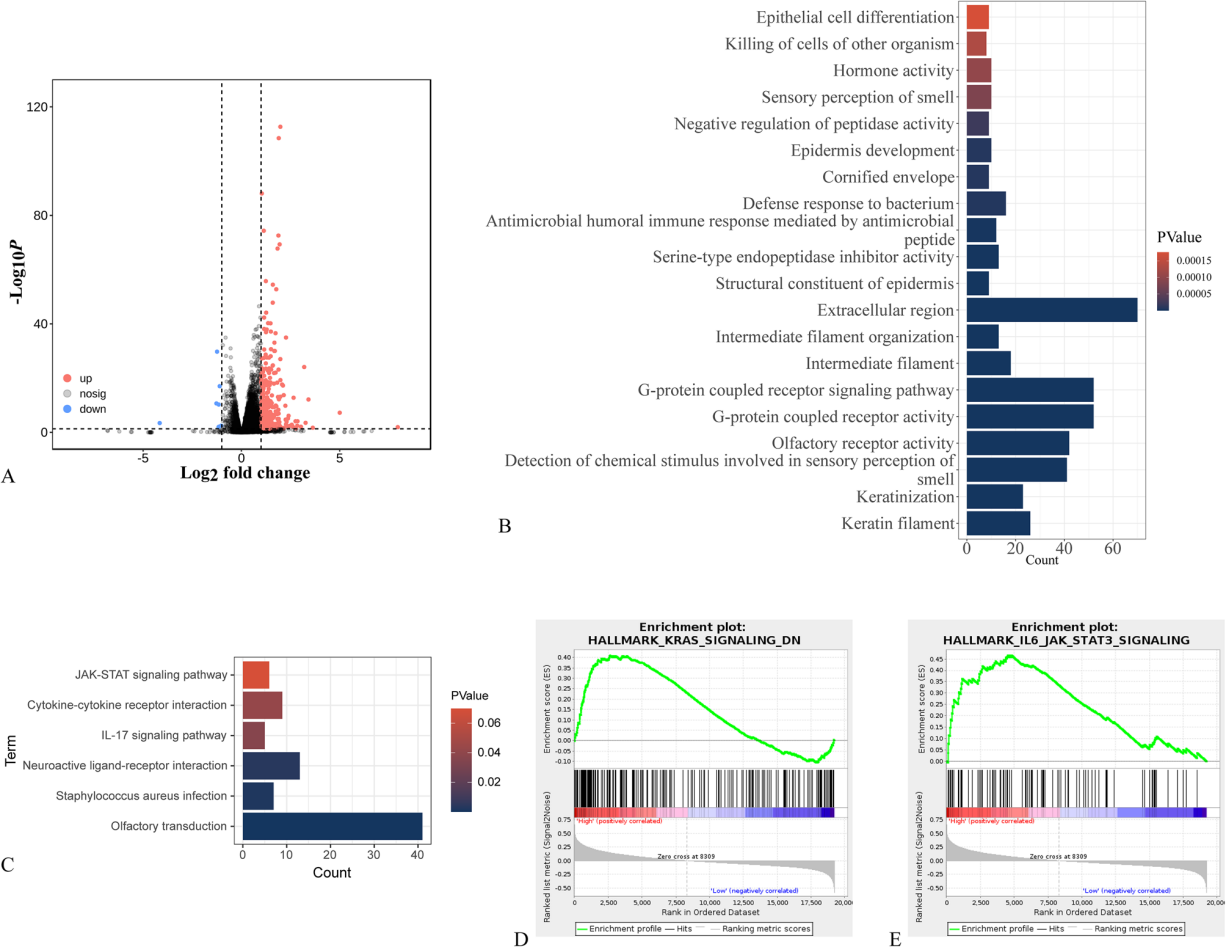
Identification and functional enrichment analysis of DEGs based on BMscore. (**A**) Volcano plot depicting DEGs between different groups. (**B**) GO enrichment analysis of BMscore-based groups. (**C**) KEGG pathway analysis of BMscore-based groups. (**D**,**E**) GSEA analysis displaying enriched signaling pathways in the high BMscore group.

Exploring the underlying mechanisms behind the involvement of BMGs in regulating the prognosis of ccRCC, a comparison of gene expression patterns between high and low BM score groups was conducted, resulting in the identification of 490 DEGs. The volcano map was employed to visualize the intergroup DEGs (Fig. 5A). The results of GO profiling indicated that these DEGs were in connection with the extracellular region and the G-protein coupled receptor signaling pathway. The results of KEGG analysis showed that the DEGs were linked to the JAK/STAT signaling pathway and the IL-17 signaling pathway (Fig. 5B,C). In comparison to the low BMscore group, the high BMscore tumor samples exhibited enrichment of genes associated with the “KRAS_SIGNALING_DN” and “IL6_JAK_STAT3_SIGNALING” pathways (Fig. 5D,E). Supplementary Table 1 and Fig. S9 present the most significantly enriched pathway identified through GSEA analysis comparing the BM score groups. These results suggest that BMGs associated with OS probably have an essential part in the ccRCC tumor microenvironment and activation of the JAK/STAT pathway. Furthermore, we utilized the STRING online database and the Cystoscope plugin “cytoHubba” to identify hub genes related to the IL6_JAK_STAT3 signaling pathway, which is associated with BMGs (Fig. S10).

### Immune cell type fractions and associated analysis in each subgroup

**Figure 6:**
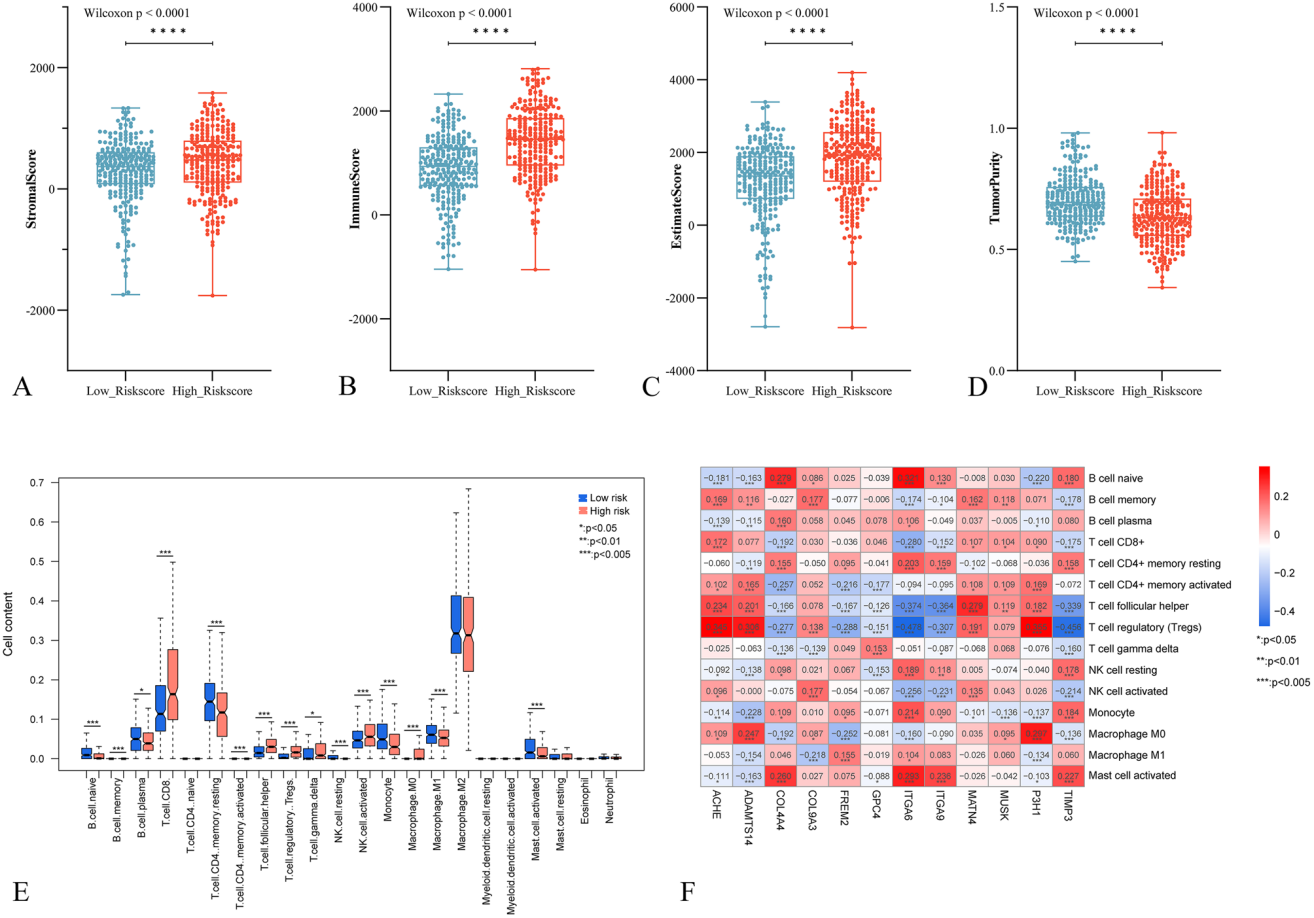
Comparison of immune cell subtyping and drug sensitivity analysis based on BMscore. (**A**–**D**) Distribution plots of various ESTIMATE scores in different risk groups. (**E**) Box plots display the proportion of different immune cell types in samples from different risk groups. (**F**) Heatmap showing the correlation between 12 prognostic-related BMGs and 15 immune cell types with significant distribution differences.

Assessing the immune profiling of different BM score groups, we applied the ESTIMATE algorithm and observed significantly higher stromal scores, immune scores, and estimate scores in the high BM score group compared to the low BM score group (Fig. 6A–C). Conversely, the high BM score group exhibited significantly lower tumor purity (Fig. 6D). These findings indicate a close association between BMGs and the tumor immune microenvironment, with the high BM score group showing increased infiltration of immune cells and stromal cells.

Furthermore, we performed immune cell profiling using CIBERSORT, which identified 15 immune cell types with significant differences in distribution. The most abundant cell types were T cell CD8+, T cell CD4+ memory resting, B cell plasma, NK cell activated, and monocyte (Fig. 6E). To explore the relationship between the 12 genes used to construct the BM score model and these 15 immune cell types, we calculated their correlations as shown in Fig. 6F.

## Discussion

Renal cell carcinoma with ccRCC histology is characterized by a high diagnostic rate, high recurrence rate, and high mortality, posing a severe threat to patients' lives and causing significant economic burden^3^. The competence of tumor cells to penetrate the BM by disintegrating BM components largely determines the prognosis of ccRCC patients^22–24^. Targeted therapy and immunotherapy have become important treatment options for ccRCC patients in current clinical practice^25,26^. BM proteins are highly conserved in multicellular organisms. Further understanding the expression patterns and mechanisms of BM proteins in ccRCC patients can help improve prognosis and develop new therapeutic targets for ccRCC^27^. In this study, we established a BM score based on BMGs. Firstly, we classified ccRCC as two subtypes according to the expression levels of 222 BMGs and compared the differential genes between the subtypes. We then conducted a cross-comparison between subtype-specific differential genes and prognosis-related BMGs, resulting in a total of 72 prognosis-related BMGs. Furthermore, using LASSO and multivariate regression analysis, we identified 12 risk-associated BMGs to construct the BM score model. This model successfully stratified ccRCC patients into two groups, and its prognostic value and accuracy were validated in two independent cohorts. Functional enrichment and immune status analysis indicated a significantly different immune microenvironment and functional states across both groups.

Previous studies have found a correlation between BMGs and the invasiveness of tumor cells, indicating their involvement in pathological processes such as tumor proliferation, metastasis, and invasion^28^. This suggests that BMGs can serve as disease biomarkers for predicting the prognosis and treatment outcomes of ccRCC. We constructed a BMscore model comprising 12 BMGs, and the prognosis between BMscore and ccRCC was significantly associated, with patients in the high BM score group exhibiting significantly lower overall survival (OS) compared to the low BM score group. The AUC values of BM score for predicting 1-year, 3-year, and 5-year OS were 0.935, 0.844, and 0.848, respectively. Furthermore, the efficacy of the BM score was validated in the verification cohort. These findings indicate that the BM score is capable of accurately predicting the prognosis of ccRCC patients, and its performance surpasses that of other BMGs-related prediction models based on the TCGA and GEO database^21,27^.

In our constructed BM score model, the expression levels of all BMGs showed significant correlation with ccRCC prognosis. Some BMGs, including ACHE^29^, ADAMTS14^30^, COL4A4^31^, MATN4^32^, P3H1^33^, and TIMP3^34^, have been found to be associated with ccRCC in previous studies. COL4A4, in particular, has been implicated in the construction of prognostic models based on BMGs in ccRCC^21,27^. COL4A4 is exclusively present in the BM and is a major structural component of the glomerular basement membrane^35^. It is located in the region 2q35-q37 with a gene span composed of 113 kb and 48 exons^35^. The composition of the BM score integrates both the subtypes of ccRCC based on BMGs and the association with prognosis. Furthermore, by incorporating analysis with clinical pathological factors, our model has been demonstrated as a reliable and independent predictor of OS. Finally, we developed a nomogram that combines the BM score with pathological stage, histologic grade, and age. This nomogram provides a pragmatic instrument for monitoring the survival of individual cases.

To elucidate the underlying mechanisms linking BMGs to tumor progression, we performed differential expression analysis based on the BM score to identify DEGs. Further functional enrichment analysis was conducted to gain insights into the biological processes involved. The results of GO analysis revealed that the DEGs were enriched in terms associated with tumor cell invasion and the BM, such as “Extracellular region,” “Serine-type endopeptidase inhibitor activity,” and “Negative regulation of peptidase activity.” Additionally, the KEGG pathway enrichment analysis showed that the DEGs were mainly involved in the JAK-STAT signaling pathway and IL-17 signaling pathway. The GSEA method identified enrichment of gene sets including “KRAS_SIGNALING_DN” and “IL6_JAK_STAT3_SIGNALING,” which aligns with the findings from KEGG enrichment analysis, suggesting a close association between the DEGs related to the BM score and the JAK/STAT3 pathway. The JAK/STAT3 pathway regulates cellular proliferation, differentiation, and apoptosis, and it has been shown to be significantly associated with ccRCC^36^. Inhibition of the JAK/STAT3 pathway can limit ccRCC progression^37^. Our findings suggest that activation of the JAK/STAT3 pathway may be one of the mechanisms underlying the progression of ccRCC with a high BM score.

Despite the clear association between BM and ccRCC established in current research, it remains unclear whether tumor immunity is regulated by BMGs. Based on the BM score, we categorized samples into high and low risk groups, previous results indicating that higher BMscore are associated with poorer prognosis. Analysis of the tumor microenvironment revealed that the high-risk group exhibited significantly higher immune infiltration and matrix cell abundance compared to the low-risk group, as indicated by stromal scores, immune scores, and estimate scores. Immune annotation analysis revealed significant differences in various immune cells between the groups, with a significantly lower abundance of resting CD4+ memory T cells observed in the high-risk group. This suggests a potential mechanism for the observed immunotherapy resistance in these patients. Additionally, there were differences in various types of T cells at the intergroup level. T cells are major participants in immune-mediated cancer control and response to immunotherapy, and BM is involved in regulating various functions of T cells^38,39^.

This study provides a preliminary exploration of the prognostic value of BMGs in ccRCC, aiming to establish a theoretical foundation for future research. However, our investigation has certain limitations. Firstly, the sample size in the validation dataset of this study is relatively small, warranting validation of BMscore with a larger independent dataset to ensure reliability. Secondly, further experiments are needed to validate the hypotheses generated in this study. We are currently undertaking prospective research to confirm our findings and plan to conduct additional foundational experiments to elucidate the value of BMGs in ccRCC more comprehensively in the future.

In summary, this study comprehensively analyzed the transcriptomic characteristics associated with the BM in ccRCC patients, elucidating the role of BMGs in ccRCC. We established and validated a risk prognostic model that can predict the survival outcomes of ccRCC patients. Furthermore, we explored the potential mechanisms underlying the progression of ccRCC associated with BMGs and investigated the differences in gene expression, functional enrichment and immune status among patients with different risk levels. Overall, this study provides valuable insights into the significance of BMGs in ccRCC and their implications for prognosis and treatment. The findings contribute to a better understanding of the molecular mechanisms underlying ccRCC and offer potential targets for personalized therapies.

## Material and methods

### Data source

Based on the Xena database (https://xenabrowser.net/datapages/), gene expression profile data related to Kidney Clear Cell Carcinoma (KIRC) was acquired. This data consists with 607 samples with gene expression values represented as standardized log (FPKM + 1, 2). Phenotype data, such as stage factors, and the survival status of the KIRC cohort, were also collected. Among the KIRC tumor samples, there were 528 samples with available clinical prognosis information. This subset of data was used as the training dataset for the current analysis.

The Gene Expression Omnibus (GEO) is a publicly curated genomic database^40^. From GEO, expression profile data for ccRCC with the accession number GSE29609 was downloaded. This dataset includes 39 ccRCC tumor samples with available clinical prognosis information. This dataset will be used as the validation dataset.

### Acquisition and subtype analysis of BMGs

Collected BMGs from the literature and obtained a total of 222 genes^41^. Consensus clustering analysis based on the expression consistency of BMGs utilized the R package “ConsensusClusterPlus”^42^, the clustering results were optimal when the value of k was set to 2. Evaluated the survival prognostic correlation among different disease subtype sample groups using the Kaplan–Meier.

### Identification and enrichment analysis of differential genes among subtypes

Based on the whole-genome expression data of KIRC samples, conducted KEGG pathway enrichment analysis among disease subtypes using the R package “GSVA”^43^. Selection of differentially expressed genes (DEGs) with R package “limma” based on comparisons between disease subtypes, with a cutoff criteria of |Log2 (fold change)| ≥ 1.0 and a P-value < 0.05.

### Selection of prognosis-associated BMGs

The median expression levels of BMGs showing significant differential expression among different subtype groups were used to divide the samples into high and low expression groups. Kaplan–Meier analysis was then employed to identify BMGs that were significantly associated with prognosis at the expression level grouping level. Univariate Cox regression analysis was conducted to select BMGs that were significantly associated with prognosis at the expression level. A significance threshold of P < 0.05 was used for gene selection. The intersection of the genes selected by both methods was visualized using a Venn diagram to identify BMGs that were significantly associated with prognosis.

### Risk model for ccRCC construct and validation

For the purpose of investigating the potential significance of BMGs in the prognosis of ccRCC patients, we applied the Least Absolute Shrinkage and Selection Operator (LASSO) method to the training set KIRC cohort for regression analysis of survival-related BMGs. Based on the LASSO regression coefficients of the selected optimized gene combination and the expression levels of target genes in the dataset, we constructed the BM score model as follows and calculated the final risk score:

$${\text{BMscore}}=\frac{ {\text{e}}^{{\text{sum (each gene's expression levels}} \times {\text{corresponding coefficient)}} }}{ {\text{e}}^{{\text{sum (each gene's mean expression levels}}\times {\text{corresponding coefficient)}} }}$$

In the KIRC training set and the validation dataset GSE29609, we calculated the BM score values for each sample using the calculation formula. The median BM score was used as the cut-off value to classify the patients into high-risk and low-risk groups. Kaplan–Meier curves were used to reflect the survival performance of the patients in each group. The predictive ability of the BM score model was evaluated using time-dependent receiver operating characteristic (ROC) curve analysis.

### Establishment of clinicopathologic nomogram

In the analysis of the KIRC training dataset, clinicopathological parameters and BM scores were integrated with the samples to identify independent prognostic clinical factors using univariate and multivariate Cox regression analysis. The identified independent prognostic factors were then combined with risk information derived from a prognostic prediction model to construct nomogram using the “rms” package^44^. Furthermore, the prediction capabilities of each factor were evaluated via the generation of ROC curves.

### Identified DEGs and enrichment analysis according to BM score

The DEG was filtered by using the R package “limma” between the high- and low-BM score groups^45^. |Log2 (fold change)| ≥ 1.0, and P-values < 0.05 were used as the cut-off criteria. Gene ontology (GO) annotation and the Kyoto Encyclopedia of Genes and Genomes (KEGG)^46^ pathway enrichment analyses were conducted using the R package “clusterProfiler”^47^. GSEA was used to identify Hallmark pathways significantly associated with BM score groups, and P value < 0.05 was chosen for the threshold of significant enrichment of associated Hallmark pathways. The GEPIA website was applied to analyze the differences in disease-free survival (DFS) and expression levels of BM score model genes between KIRC and control groups^48^. And we utilize the GEPIA database to assess the expression profile of BM score model genes in normal tissues and other tumor tissues. The immunohistochemical staining of prognostic genes was obtained from The Human Protein Atlas^49^ (https://www.proteinatlas.org/). The immunohistochemical intensity results were obtained from the HPA website.

### Potential impact of BM score on the immune microenvironment of ccRCC

Estimate the tumor purity of KIRC samples using the “ESTIMATE” R package^50^. Employ CIBERSORT to assess the degree of immune cell infiltration among different BM score groups, including the proportions and CIBERSORT indices of 22 tumor-infiltrating immune cells, and visualize the results through bar plots and heatmaps^51^. Utilize Chi-square tests to determine if there are any significant differences among the groups based on the associated scores.

### Protein–protein interaction and identification of hub genes

Protein–protein interaction (PPI) predictions were retrieved from the STRING database^52^. The obtained PPI network was visualized using the Cytoscape software, and the “cytohubba” plugin was employed for modular analysis^53^. The top 10 hub genes were identified, defined as genes that are associated with the expression of other genes^54^.

### Supplementary Information

Supplementary Information.

## Data Availability

All the data used in this study were publicly available at The Cancer Genome Atlas portal (TCGA, https://portal.gdc.cancer.gov/) and Gene Expression Omnibus (GEO, https://www.ncbi.nlm.nih.gov/ geo/).

## Abbreviations

AUC: Area under the curve
BM: Basement membrane
BMGs: BM-related genes
ccRCC: Clear cell renal cell carcinoma
DEGs: Differential expression genes
FDR: The false discovery rate
GEO: Gene expression omnibus
GO: Gene ontology
IC50: Half-maximal inhibitory concentration
KEGG: Kyoto encyclopedia of genes and genomes
KIRC: Kidney clear cell carcinoma
LASSO: Least absolute shrinkage and selection operator
MMPs: Matrix metalloproteinases
NES: Normalized enrichment score
NOM p-val: Nominal P value
OS: Overall survival
PPI: Protein–protein interaction
ROC: Receiver operating characteristic
