# Supplementary material for: Integration of basement membrane-related genes in a risk signature for prognosis in clear cell renal cell carcinoma
Source: Sci Rep. 2024 Feb 16;14:3893. doi: 10.1038/s41598-024-54073-1 (PMC10873511; doi:10.1038/s41598-024-54073-1)
Supplement: Supplementary file 1 — Supplementary Information. [file 41598_2024_54073_MOESM1_ESM.docx]

**Integration of Basement Membrane-Related Genes in a Risk Signature for Prognosis in Clear Cell Renal Cell Carcinoma**

Bowen Xia^1,2*^, Jingwei Wang^3*^, Dongxu Zhang^1,2^, Xiaopeng Hu^1,2#^

^1^Department of Urology, Beijing Chao-Yang Hospital, Capital Medical University, Beijing, China.

^2^Institute of Urology, Capital Medical University, Beijing, China.

^3^Department of Occupational Medicine and Toxicology, Clinical Center for Interstitial Lung Diseases, Beijing Institute of Respiratory Medicine, Beijing Chaoyang Hospital, Capital Medical University, Beijing 100020, China

^*^These authors equally contributed to this work and should be regarded as co-first authors.

^#^Corresponding author contact information:

Professor Xiaopeng Hu, Department of Urology, Beijing Chao-Yang Hospital, Capital Medical University, Beijing, China.

Add: No.8 Worker’s Stadium, Chaoyang District, 100020, Beijing, China;

E-mail: xiaopeng_hu2023@126.com

**Supplementary materials**


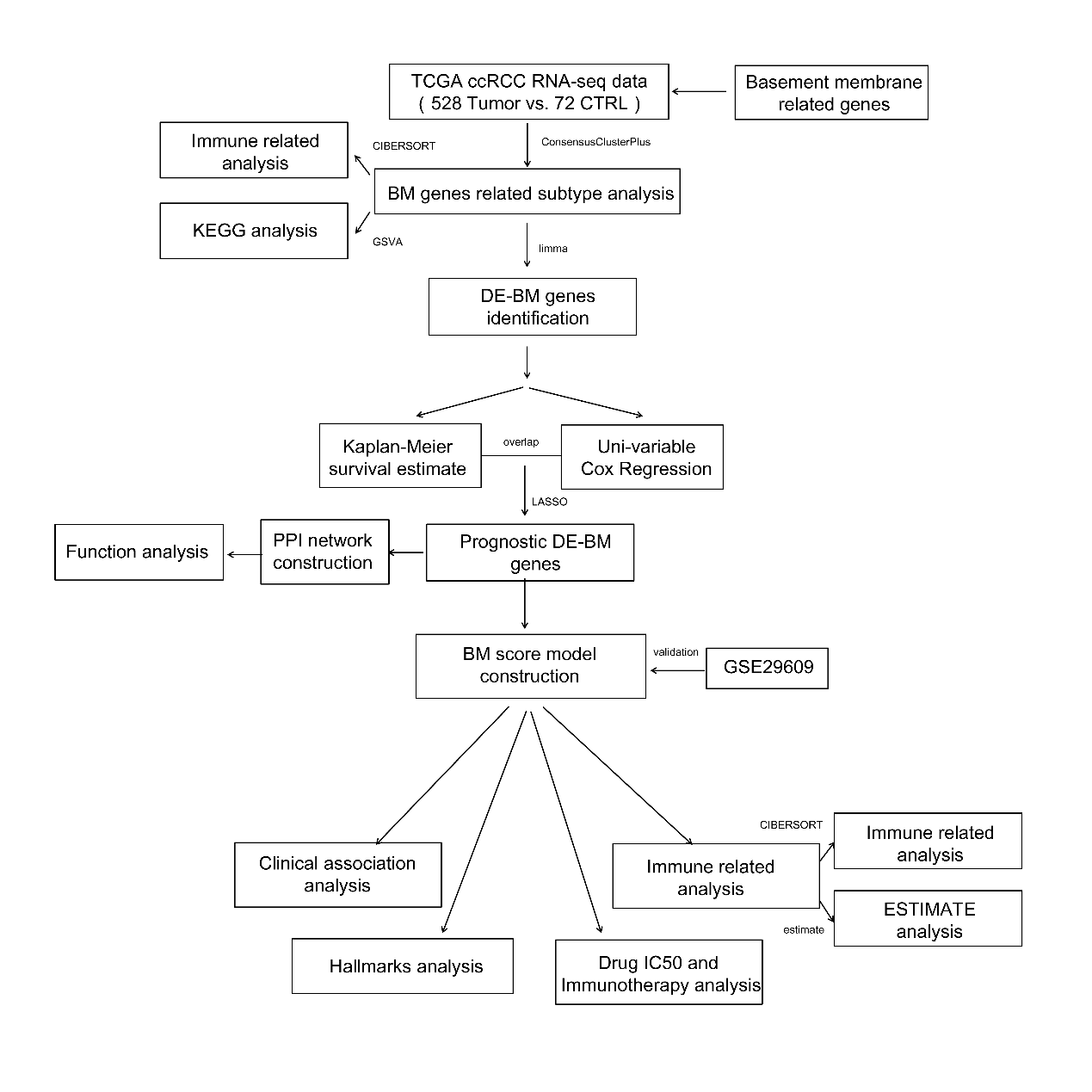


**Figure S1:** Flow chart of the current study.


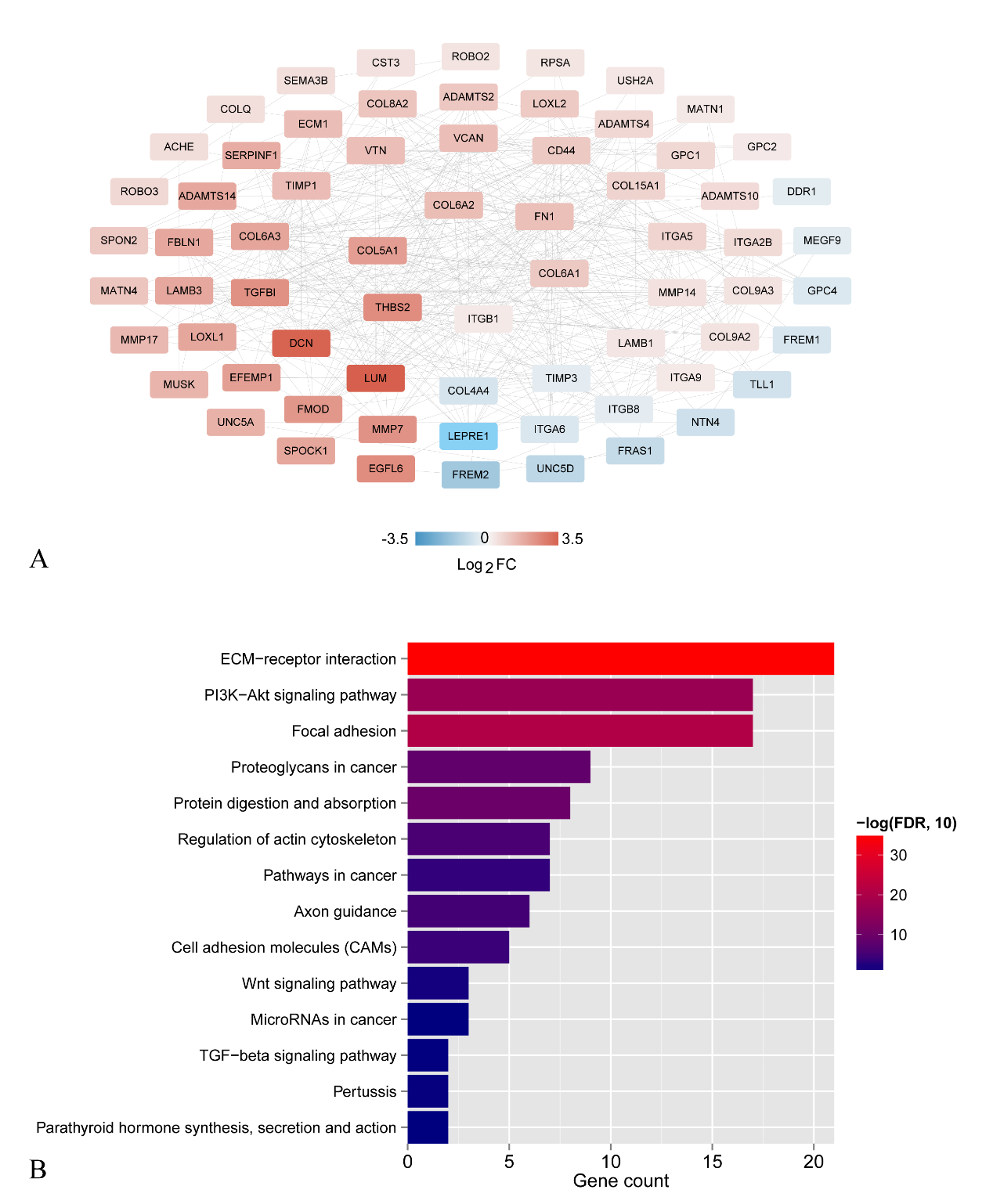


**Figure S2:** (A) Protein-Protein Interaction (PPI) network diagram of prognostic-related BMGs. Colors represent the degree of significant differences. (B) Bar plot of significantly correlated KEGG signaling pathways associated with prognostic-related BMGs. The x-axis represents the number of genes, the y-axis represents the pathway names, and the color represents the significance.


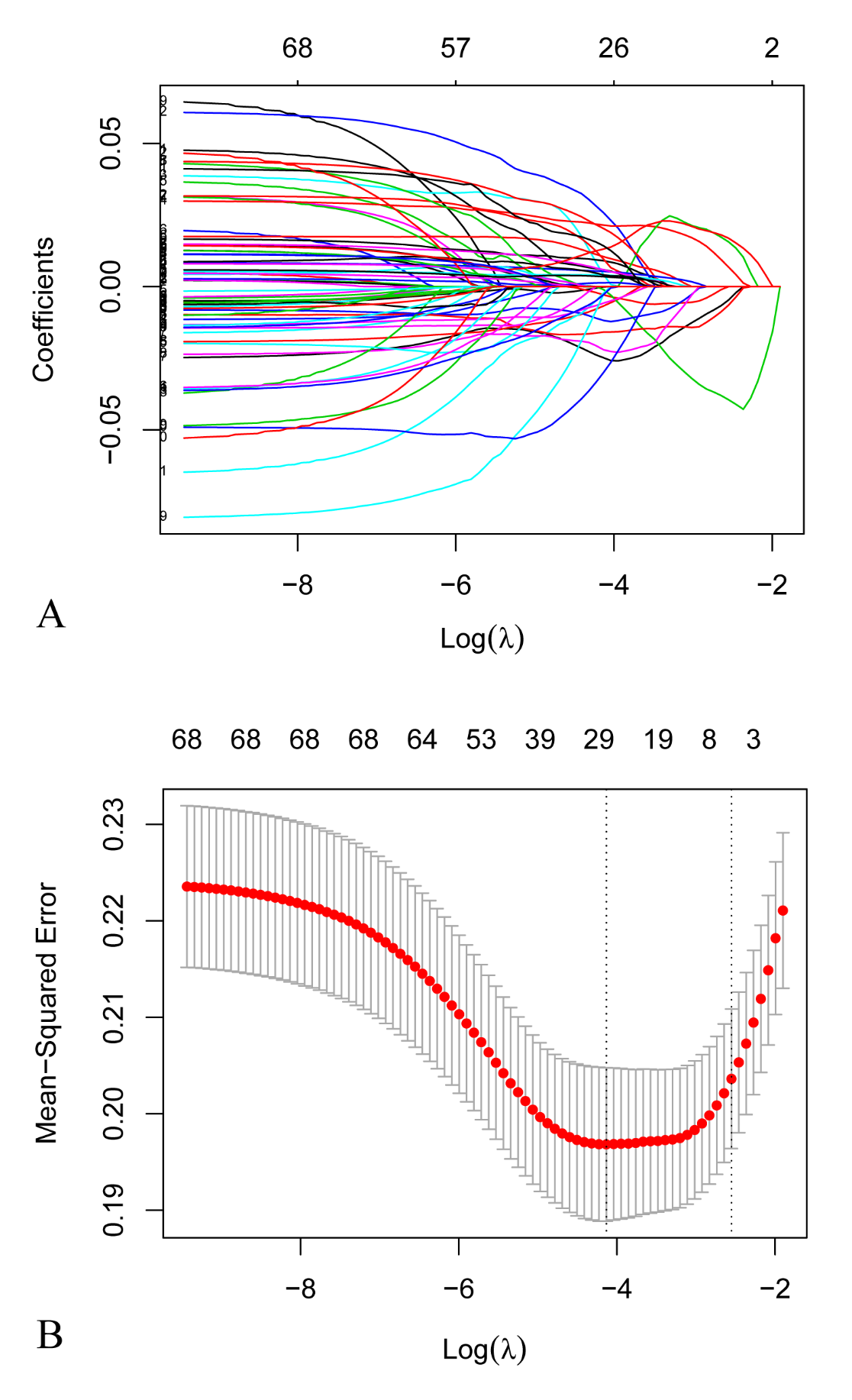


**Figure S3:** (A-B) Selection of OS-related BMGs by LASSO analysis.


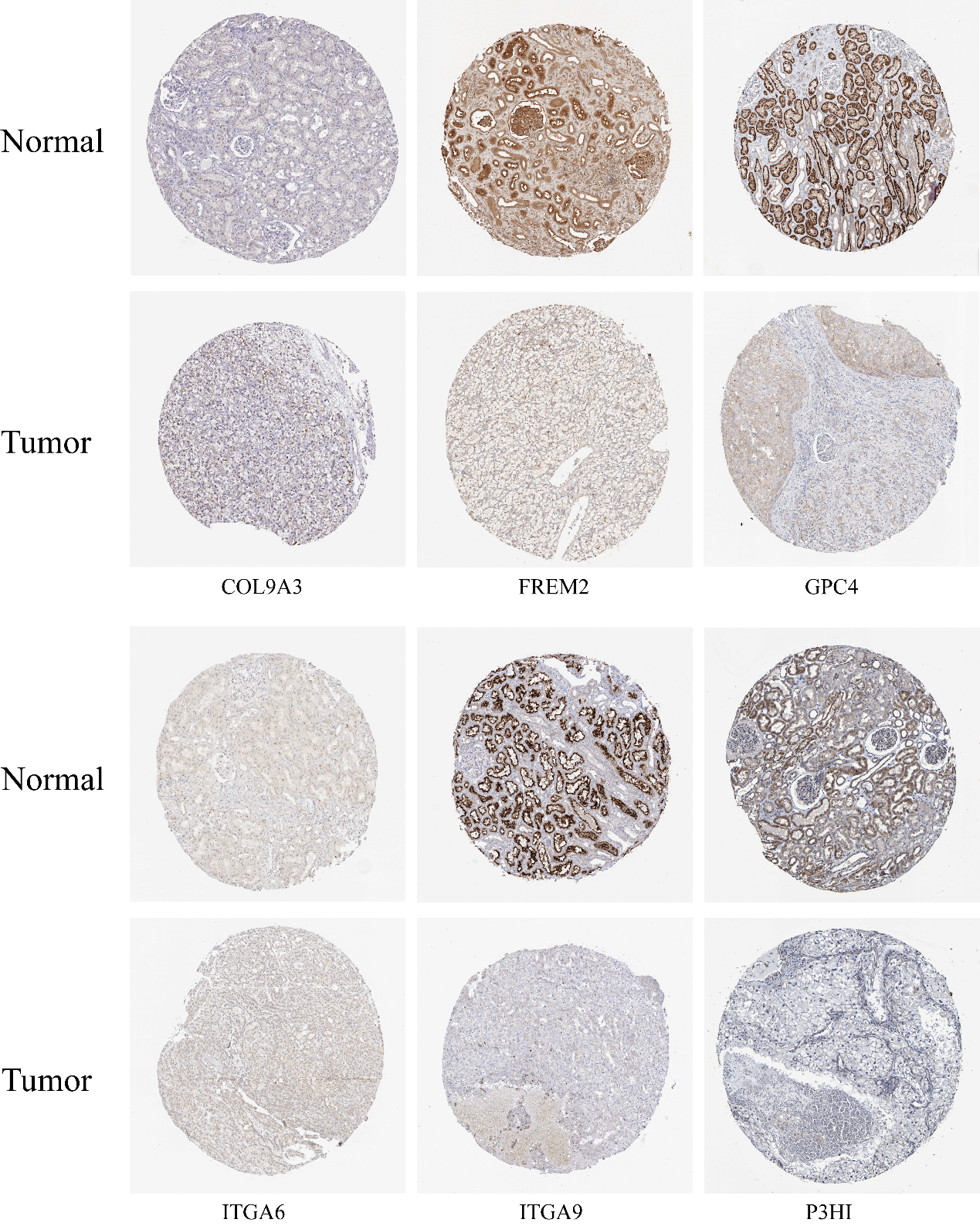


**Figure S4:** Immunohistochemical staining analysis of prognostic genes in renal cancer tissues from The Human Protein Atlas.


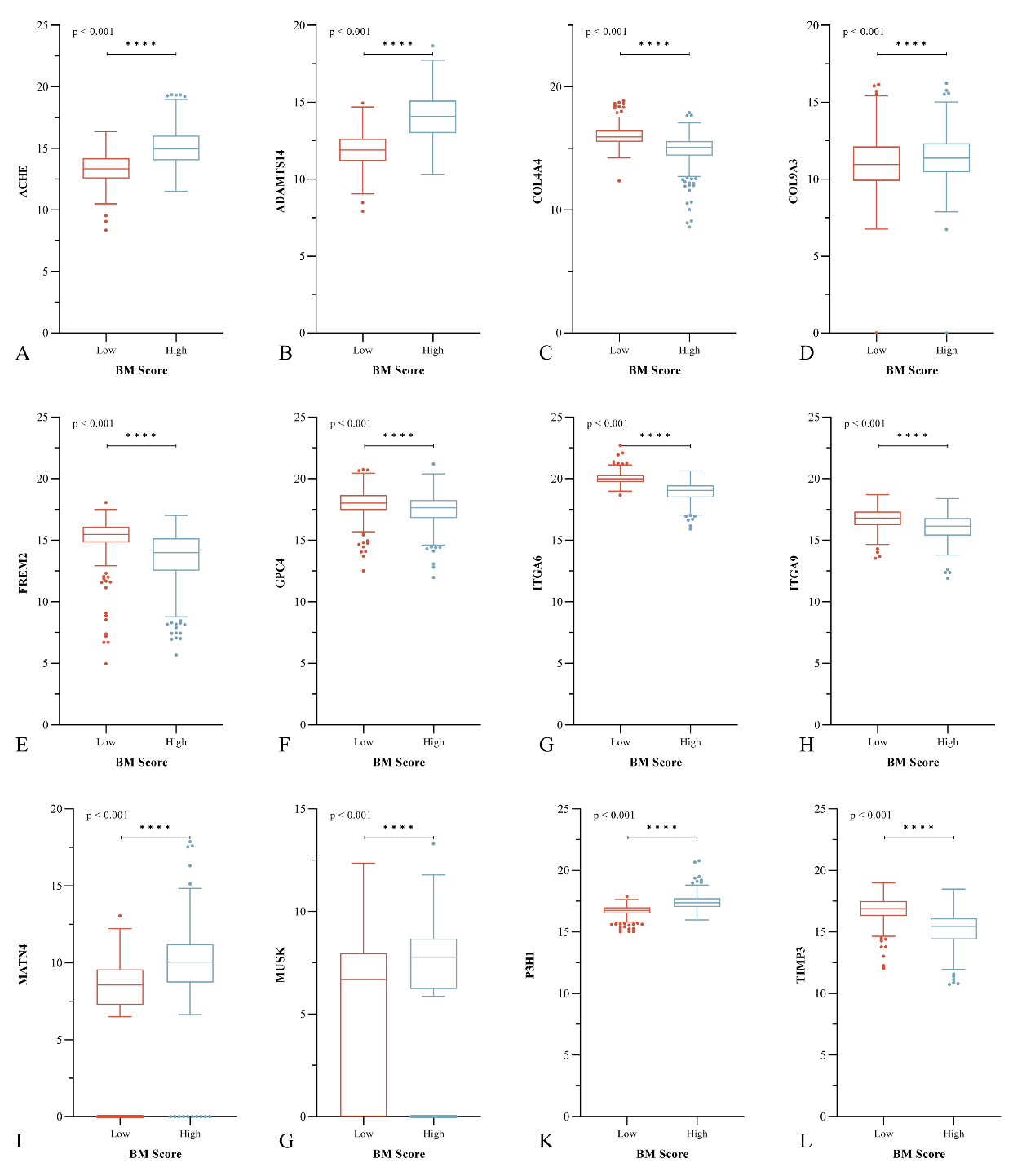


**Figure S5**: (A-L) Expression levels of 12 OS-related BMGs.


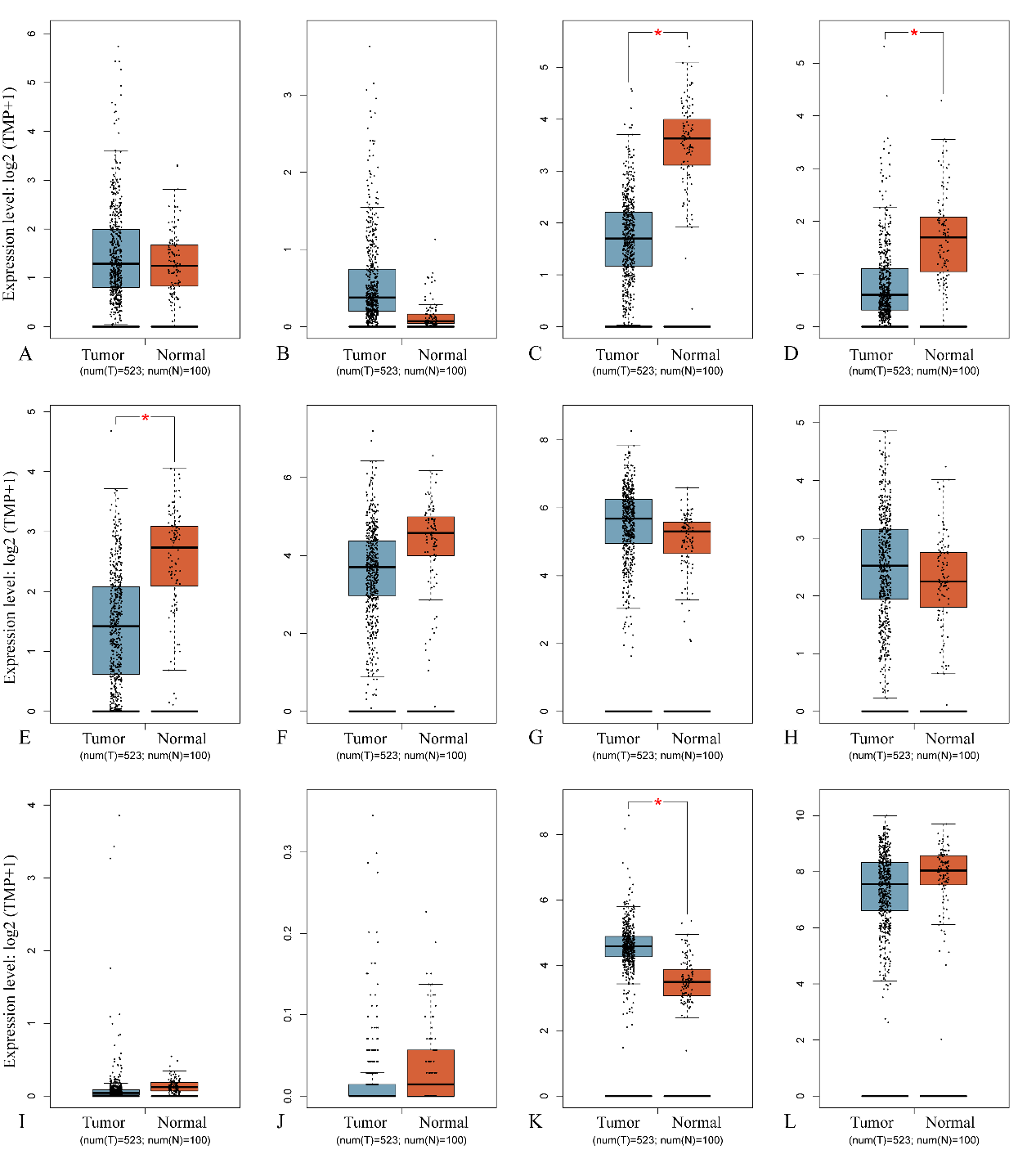


**Figure S6:** The expression of OS-related BMGs between normal renal tissues and ccRCC samples from GEPIA (normal tissues include data from TCGA and GTEx database). *Represents statistical differences. Red color represents tumor samples and gray color represents normal samples. ccRCC clear cell renal cell carcinoma.


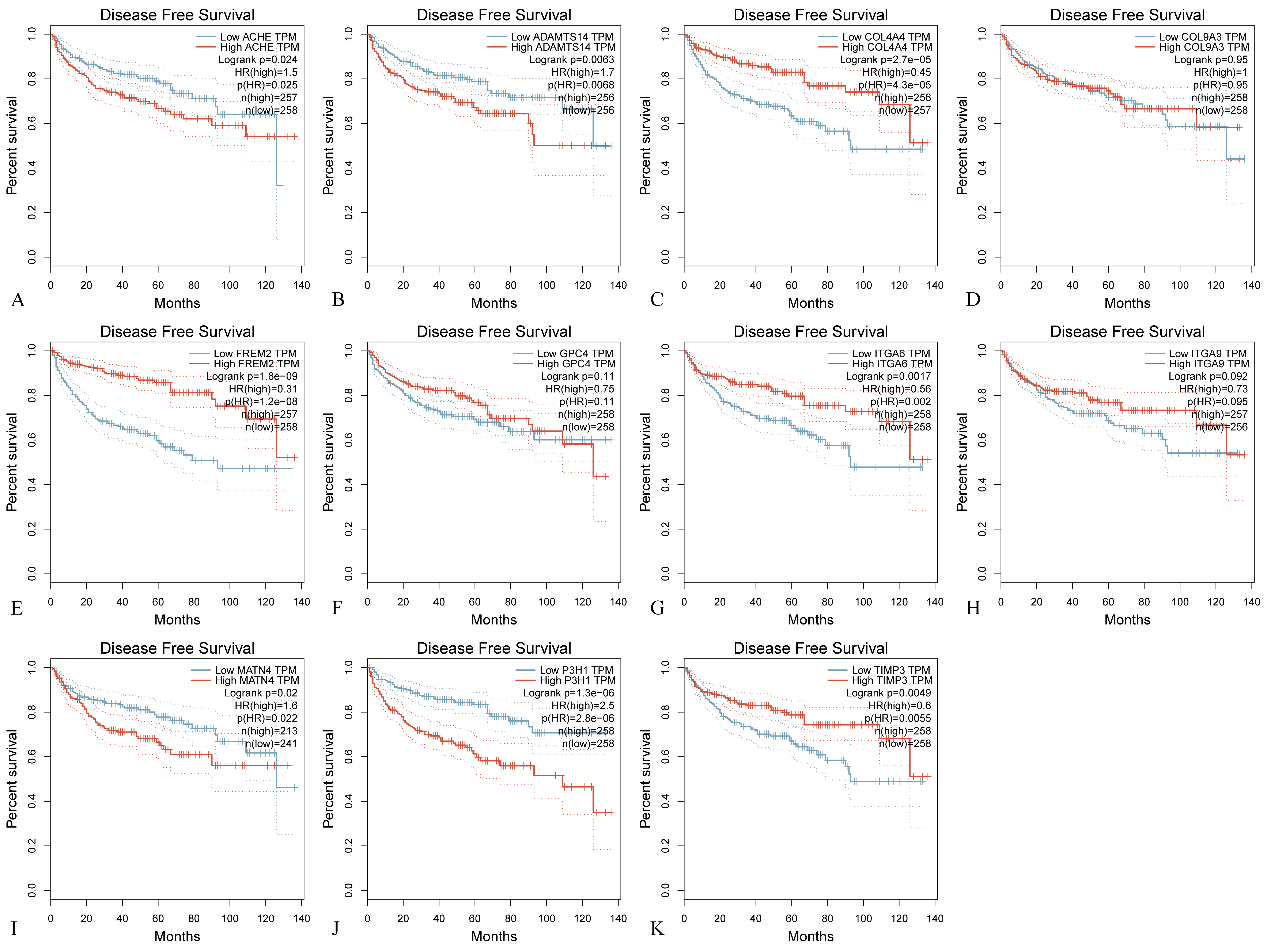


**Figure S7:** Validation of the BMGs signature. (A-K) The correlation between OS-related BMGs’ expression and the DFS of ccRCC got from GEPIA. ccRCC clear cell renal cell carcinoma.


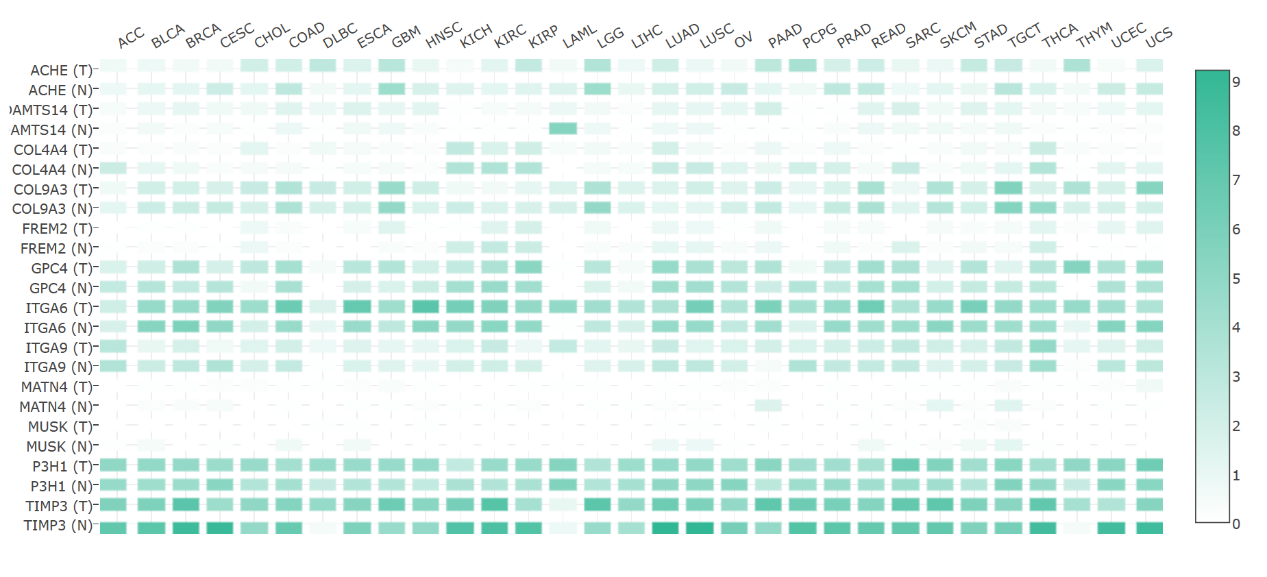


**Figure S8:** The expression profile of the BMGs signature in normal tissues and other tumor tissues, with darker colors indicating higher expression levels. "N" denotes normal tissues, while "T" represents tumor tissues. ACC, Adrenocortical carcinoma; BLCA, Bladder Urothelial Carcinoma; BRCA, Breast invasive carcinoma; CESC, Cervical squamous cell carcinoma and endocervical adenocarcinoma; CHOL, Cholangiocarcinoma; COAD , Colon adenocarcinoma; DLBC, Lymphoid Neoplasm Diffuse Large B-cell Lymphoma; ESCA, Esophageal carcinoma; GBM, Glioblastoma multiforme; HNSC, Head and Neck squamous cell carcinoma; KICH, Kidney Chromophobe; KIRC, Kidney renal clear cell carcinoma; KIRP, Kidney renal papillary cell carcinoma; LAML, Acute Myeloid Leukemia; LGG, Brain Lower Grade Glioma; LIHC, Liver hepatocellular carcinoma; LUAD, Lung adenocarcinoma; LUSC, Lung squamous cell carcinoma; OV, Ovarian serous cystadenocarcinoma; PAAD, Pancreatic adenocarcinoma; PCPG, Pheochromocytoma and Paraganglioma; PRAD, Prostate adenocarcinoma; READ, Rectum adenocarcinoma; SARC, Sarcoma; SKCM, Skin Cutaneous Melanoma; STAD, Stomach adenocarcinoma; TGCT, Testicular Germ Cell Tumors; THCA, Thyroid carcinoma; THYM, Thymoma; UCEC, Uterine Corpus Endometrial Carcinoma; UCS, Uterine Carcinosarcoma;


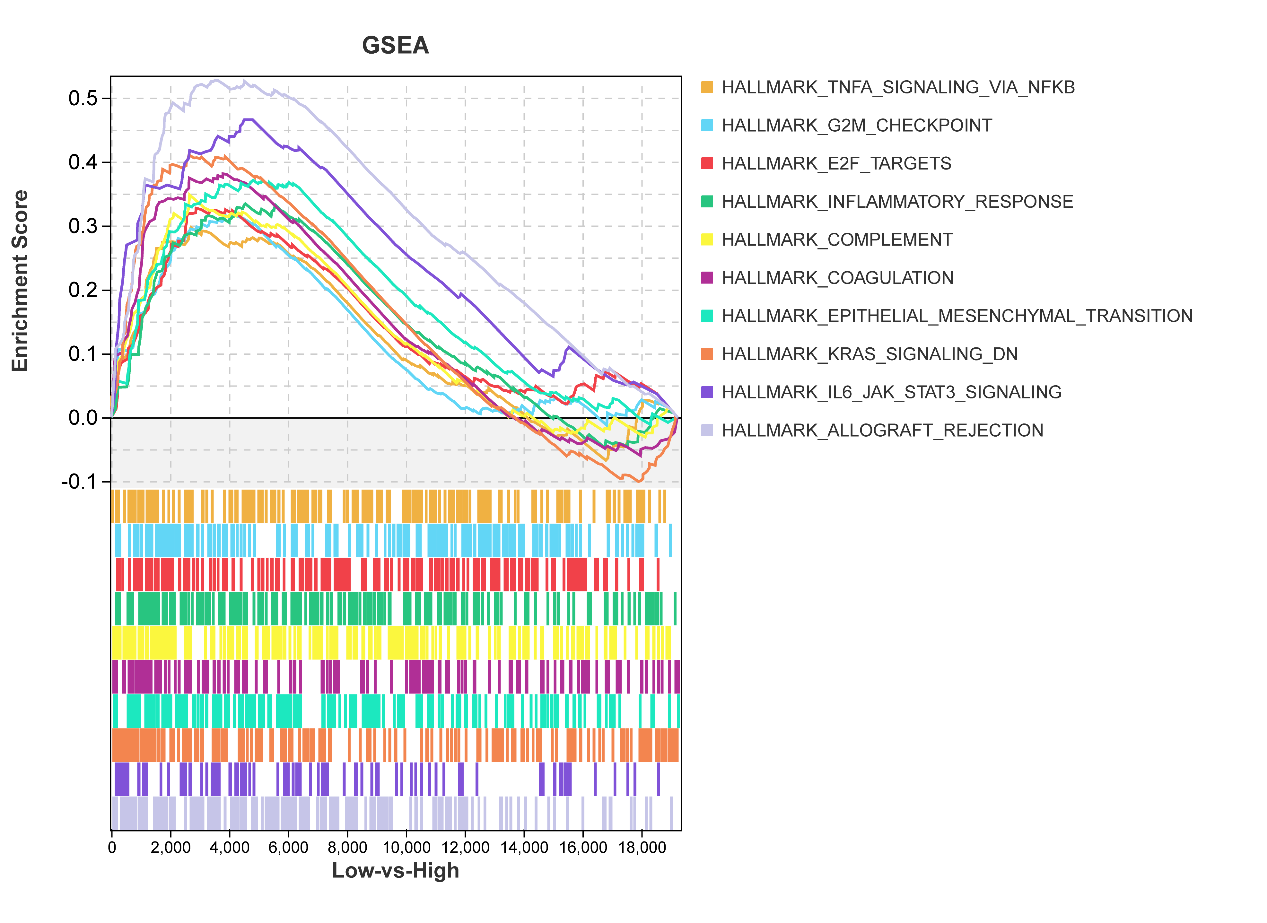


**Figure S9:** Top 10 pathways for GSEA enrichment analysis with high and low BMscore groups.


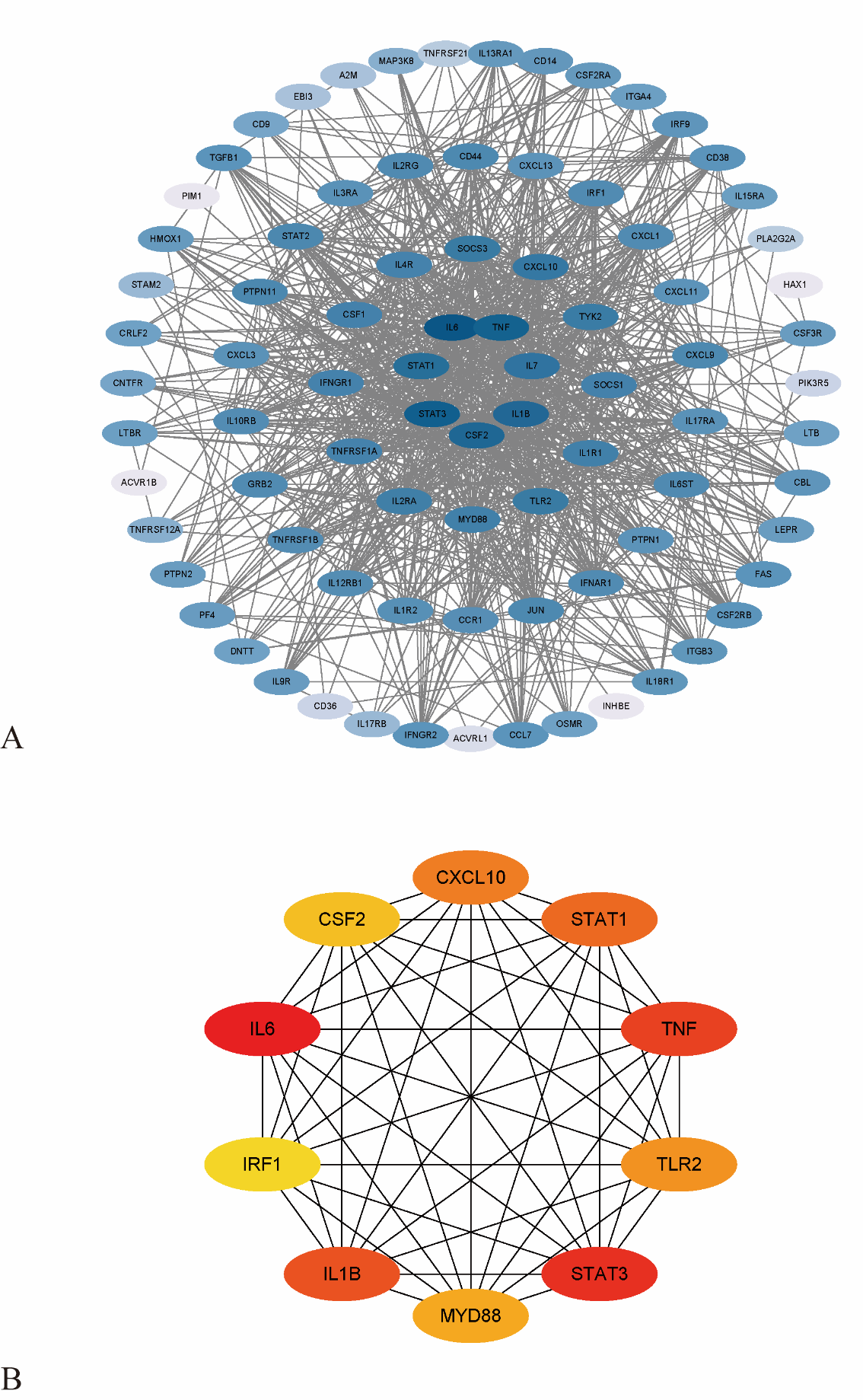


**Figure S10:** (A) Using the STRING online database, the core genes PPI network was constructed. (B) Top 10 hub genes from the core genes ranked by ‘CytoHubba’ methods.

**Table S1** HALLMARK pathway enrichment analysis by GSEA.

| HALLMARK pathway names | NES | NOM p-val | FDR |
| --- | --- | --- | --- |
| ALLOGRAFT_REJECTION | 0.527 | <0.001 | <0.001 |
| IL6_JAK_STAT3_SIGNALING | 0.465 | <0.001 | <0.001 |
| KRAS_SIGNALING_DN | 0.410 | <0.001 | <0.001 |
| EPITHELIAL_MESENCHYMAL_TRANSITION | 0.371 | <0.001 | 0.011 |
| COAGULATION | 0.381 | 0.001 | 0.014 |
| COMPLEMENT | 0.347 | <0.001 | 0.026 |
| INFLAMMATORY_RESPONSE | 0.334 | 0.002 | 0.043 |
| E2F_TARGETS | 0.327 | 0.009 | 0.051 |
| G2M_CHECKPOINT | 0.317 | 0.018 | 0.078 |
| TNFA_SIGNALING_VIA_NFKB | 0.297 | 0.042 | 0.139 |

NES: normalized enrichment score; NOM p-val: Nominal P Value; FDR: The false discovery rate.
